# Simultaneous Measurement of Temperature and Mechanical Strain Using a Fiber Bragg Grating Sensor

**DOI:** 10.3390/s20154223

**Published:** 2020-07-29

**Authors:** Shiuh-Chuan Her, Wei-Nan Lin

**Affiliations:** Department of Mechanical Engineering, Yuan Ze University, Chung-Li 320, Taiwan; s975029@mail.yzu.edu.tw

**Keywords:** fiber Bragg gratin, Bragg wavelength shift, temperature-strain discrimination

## Abstract

Based on the shift of the Bragg wavelength, fiber Bragg grating (FBG) sensors have been employed to measure a variety of physical parameters such as stress, strain, displacement, temperature, vibration and pressure. In this work, a simple and easy way to be implemented FBG sensing methodology was proposed to measure the temperature and strain simultaneously. Half of the FBG was bonded on the host structure, while the other half of the FBG was left free. The host structure was an aluminum test specimen with dimensions of 20 × 3.8 × 0.5 ${\mathrm{cm}}^{3}$. As the host structure subjected to mechanical and thermal loadings, the Bragg wavelengths reflected from the bonded and unbonded FBGs are different. Theoretical predictions of the Bragg wavelength shifts of the bonded and unbonded FBGs were presented. Utilizing the Bragg wavelength shift of unbonded FBG, the temperature can be determined and is independent of mechanical strain. The Bragg wavelength shift of the bonded FBG allows the determination of the mechanical strain. The temperature measured by FBG sensor was compared with the result from a thermocouple, while the mechanical strain was validated with the theoretical prediction. Good agreement between the experimental measurement and theoretical prediction demonstrates that temperature-strain discrimination can be realized using the proposed method with one single FBG sensor.

## 1. Introduction

Structural health monitoring (SHM) has been widely utilized in civil structures such as bridges, pipelines, dams and tunnels, to maintain operational safety and extend service life by enhancing its durability and reliability. Fiber optic sensors (FOS) with small size, light weight, immunity to electromagnetic interference (EMI) and corrosion and embedding capability have demonstrated their inherent advantages for integration in civil structures. In recent years, a great number of novel sensing technologies based on fiber optic sensors have been proposed for structural health monitoring. FOS exhibits several advantages over conventional sensors such as high sensitivity, long distance, multiplexing and distributed monitoring. It has the capability for the measurements of strain, displacement and temperature over the entire length of the fiber. In order to measure distributed strains over the entire length of a fiber, several techniques have been proposed such as grating based sensors (fiber Bragg grating, FBG), reflectometry-based sensors (Rayleigh and Brillion scattering) and interferometry based sensors (Fabry-Perot and Mach-Zehnder) [1,2]. Reflectometry sensors based on the principle of Optical Time-Domain Reflectometry (OTDR) are suitable for long distance and continuous measurements [3,4]. The FBG sensor is based on the shift of a Bragg wavelength due to the variations of temperature, strain and vibration in the surrounding environment [5]. The Fabry-Perot sensor utilizes the intensity of the interference signals illuminated between two parallel reflecting interfaces to measure the temperature and pressure [6]. The Mach-Zehnder sensor is based on the intensity of the phase shift variations illuminated between two beams split from a single light source [7].

FBG sensors have numerous advantages for application in structural health monitoring, such as light weight, multiple sensors in one fiber, tolerance in hostile environments (temperature, chemical components), long-term stability and durability. Grooteman [8] proposed a new damage indicator to detect a load path failure for a multiple load path structure, based on the strain response measured by FBG sensors. Yu and Okabe [9] employed FBG acoustic emission sensor to identify damage localization in carbon fiber reinforced plastic (CFRP) laminates. Jacobsz and Jahnke [10] utilized FBG sensor to detect the leak of water pipelines. Tsukada et al. [11] evaluated the residual stress arose in thick thermoplastic composite laminates during high-rate manufacturing processes using FBG sensors. Jang and Kim [12] utilized FBG sensors to determine the impact location on a composite stiffened panel using a triangulation method. Anastasopoulos et al. [13] proposed a novel vibration-based method using FBG sensors to identify the damage in civil structures for structural health monitoring. Lv et al. [14] used two FBG sensors to simultaneously measure the flow rate and temperature in a pipeline. Górriz et al. [15] presented a new FBG sensor embedded in concrete structures to monitor temperature during fires. Weisz-Patrault et al. [16] proposed a new roll-gap friction sensor based on FBG strain measurement to evaluate the contact stress during cold rolling process.

In many engineering applications, simultaneous measurement of strain and temperature are highly desired, such as operating in aircraft structures [17] and wind turbine blades [18] and process control of petrochemical plants [19]. Most of the fiber optic sensors used for SHM are sensitive to both strain and temperature, leading to an unwanted cross-sensitivity between these two parameters [20]. One of the applicable approaches used for the discrimination between the strain and temperature is to incorporated different sensing techniques, such as combining an FBG sensor with Raman scattering [19]. Bolognini et al. [21] integrated Raman and Brillouin scattering to measure strain and temperature simultaneously. Zou et al. [22] utilized Brillouin frequency shift and birefringence to distinguish between the strain and temperature. Lu et al. [23] proposed a method combining the optical time-domain reflectometry (OTDR) and birefringence measurements to evaluate the temperature and strain. Zhu et al. [24] presented a novel sensing concept based on an optical fiber sensor embedded in laminated composites for simultaneous measurement of the temperature and strain. However, all of the approaches mentioned above can lead to a significant increase of the cost and complexity of the sensing system.

In this work, a simple and easy way to implement method based on one single FBG sensor was proposed to measure the strain and temperature simultaneously. Half of the FBG was bonded on the hos structure, while the other half of the FBG was left free. The host structure was an aluminum test specimen with dimensions of 20 × 3.8 × 0.5 ${\mathrm{cm}}^{3}$. As the specimen subjected to mechanical and thermal loadings, the Bragg wavelengths reflected from the bonded and unbonded FBGs are different. Strain and temperature discrimination can be achieved based on the Bragg wavelengths reflected from the bonded and unbonded parts of FBG. Experimental measurements were verified with the theoretical prediction.

## 2. FBG Sensing Principle for Temperature and Strain

A fiber Bragg grating is a periodic modulation of the refractive index formed by exposure to UV radiation along the fiber core over a limited length of the fiber. As the broad band light travels through an optical fiber, the grating will serve as a narrow band filter to reflect a single-peak wavelength known as Bragg wavelength. The Bragg wavelength is given by:

(1) $$\lambda_{B}=2n\Lambda$$

where $\lambda_{B}$ denotes the Bragg wavelength at the initial state reflected from the FBG, *n* is the effective refractive index and Λ is the grating period.

The Bragg wavelength could be shifted due to the variations of temperature ΔT and mechanical strain ε in the optical fiber as follows [25,26,27]:

(2) $$\frac{\Delta \lambda_{B}}{\lambda_{B}}=k_{\varepsilon }\varepsilon +k_{T}\Delta T$$

$$k_{\varepsilon }=1-\frac{1}{2}n_{0}^{2}\left[\left(1-\upsilon_{f}\right)p_{12}-\upsilon_{f}p_{11}\right]$$

$$k_{T}=\left[1-\frac{1}{2}n_{0}^{2}\left(p_{11}+2p_{12}\right)\right]\alpha_{f}+\frac{\xi }{n_{0}}$$

where $k_{\varepsilon }$ and $k_{T}$ represent the strain coefficient and temperature coefficient of FBG sensor, respectively [26], $n_{0},\;\upsilon_{f},\;\alpha_{f}\;\mathrm{and}\;\xi$ are refractive index, Poisson’s ratio, coefficient of thermal expansion and thermo-optic coefficient, respectively, and $p_{11}\;\mathrm{and}\;p_{12}\;$denote Pockel’s constants.

The sensing principle of FBG sensor is based on the shift of Bragg wavelength as shown in Equation (2) to measure the mechanical strain and temperature.

### 2.1. Bragg Wavelength Shift Due to the Temperature Change

For an FBG sensor partially bonded on a host structure, the shifts of Bragg wavelengths from the unbonded and bonded parts of FBG induced by a temperature change ΔT are different. The shift of Bragg wavelength from the unbonded part of FBG is readily to be determined by substituting the temperature change ΔT into Equation (2):

(3) $$△\lambda_{B}=k_{T}\Delta T\lambda_{B}=\left\{\left[1-\frac{1}{2}n_{0}^{2}\left(p_{11}+2p_{12}\right)\right]\alpha_{f}+\frac{\xi }{n_{0}}\right\}\Delta T\lambda_{B}$$

The shift of the Bragg wavelength from the bonded part of FBG induced by a temperature change can be attributed to the temperature change Δ*T* and the thermal strain $\varepsilon_{T}\;$due to the mismatch of the thermal expansion between the optical fiber and host structure. The thermal strain derived by Her and Huang [27] is adopted and presented as follows:

(4) $$\varepsilon_{T}=\frac{\left(\alpha_{h}-\alpha_{f}\right)\Delta T}{E_{f}\left(\frac{\pi r_{f}^{2}}{2hr_{p}E_{h}}+\frac{1}{E_{f}}\right)}\left[1-\frac{\cosh\left(\lambda_{1}x\right)}{\cosh\left(\lambda_{1}L_{f}\right)}\right]+\alpha_{f}\Delta T$$

$$\lambda_{1}=\sqrt{\left[\frac{2r_{p}}{\pi r_{f}^{2}}\left(\frac{\pi r_{f}^{2}}{2hr_{p}E_{h}}+\frac{1}{E_{f}}\right)\int_{0}^{cos^{-1}\left(\frac{b}{r_{p}}\right)}\frac{1}{\frac{r_{p}\left(1-sin\theta \right)}{G_{a}}+\frac{r_{p}}{G_{p}}ln\left(\frac{r_{p}}{r_{f}}\right)}d\theta \right]}$$

where α, r, *E* and *G* represent the coefficient of thermal expansion, radius, Young’s modulus and shear modulus, respectively; subscripts *h, f, p* and *a* denote the host structure, optical fiber, protective coating and adhesive, respectively. Numerical values of the material properties for the optical fiber, protective coating, adhesive and host structure used in this work are listed in Table 1.

The shift of Bragg wavelength from the bonded part of FBG due to the temperature change can be obtained by substituting the thermal strain $\varepsilon_{T}$ as shown in Equation (4) and temperature change ΔT into Equation (2) yields:

(5) $$\Delta \lambda_{B}=\left[k_{\varepsilon }\varepsilon_{T}+k_{T}\Delta T\right]\lambda_{B}$$

### 2.2. Bragg Wavelength Shift Due to the Mechanical Strain

For an FBG sensor bonded on a host structure, the strain transferred from the host structure to the optical fiber is dependent on the bonding characteristics such as adhesive, protective coating and bonding length. The mechanical strain transferred to the FBG sensor from the host structure derived by Her and Huang [28] is adopted and presented as follows:

(6) $$\varepsilon_{M}=\frac{\varepsilon_{0}}{E_{f}\left(\frac{\pi r_{f}^{2}}{2hr_{p}E_{h}}+\frac{1}{E_{f}}\right)}\left[1-\frac{cosh\left(\lambda_{1}x\right)}{cosh\left(\lambda_{1}L_{f}\right)}\right]$$

where $\varepsilon_{0}$ is the mechanical strain applied on the host structure.

The Bragg wavelength shift of the FBG bonded on the host structure can be determined by substituting the mechanical strain Equation (6) into Equation (2), which leads to:

(7) $$\Delta \lambda_{B}=k_{\varepsilon }\varepsilon_{M}\lambda_{B}$$

### 2.3. Bragg Wavelength Shift Due to the Temperature Change and Mechanical Strain

Utilizing the principle of superposition, the Bragg wavelength shift of the FBG bonded on the host structure subjected to both temperature change and mechanical strain can be obtained by summing Equations (5) and (7) as follows:

(8) $$\Delta \lambda_{B}=\left[k_{\varepsilon }\varepsilon_{T}+k_{T}\Delta T\right]\lambda_{B}+k_{\varepsilon }\varepsilon_{M}\lambda_{B}$$

## 3. Experimental Measurements of Temperature and Strain

**Figure 1:**
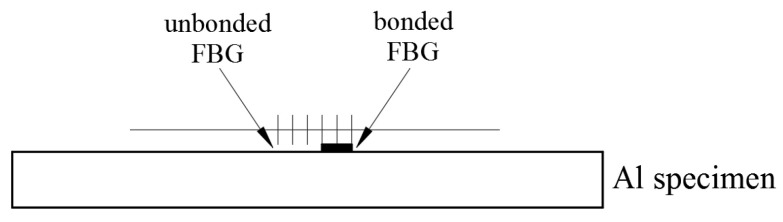
A schematic diagram of bonded and unbonded fiber Bragg grating (FBG) on the Al specimen.

The theoretical predictions of the Bragg wavelength shifts for an FBG sensor bonded on the host structure subjected to thermal and mechanical loadings were presented in the previous section. On the basis of the theoretical analysis, a sensing methodology was proposed to achieve the simultaneous measurement of temperature and strain. In this work, an FBG sensor with grating length of 40 mm was adhered to the central area of an aluminum test specimen with dimensions of 20 × 3.8 × 0.5 ${\mathrm{cm}}^{3}$ using epoxy. In order to simultaneously detect the temperature and mechanical strain, half of the FBG sensor was bonded on the specimen and the other half of FBG sensor was left free. A schematic diagram to illustrate the bonded and unbonded FBG on the Al test specimen is presented in Figure 1. Utilizing the Bragg wavelengths reflected from the bonded and unbonded parts of the FBG sensor, discrimination between the temperature and strain was realized. The FBG sensing system consists of a broad band light source (ASE 1550A, Faztec Co., New Taipei City, Taiwan) and an optical spectrum analyzer (AQ 6331, Ando Electric Co., Tokyo, Japan). As the broad band light transmits into an optical fiber, a narrow band spectrum named Bragg wavelength is reflected from the FBG sensor. In the transmitted light, the Bragg wavelength is missing. Thus, the FBG sensor acts as a narrow band filter. The reflected Bragg wavelength is affected by both the temperature and strain and can be detected by an optical spectrum analyzer (OSA). In this work, the Bragg wavelength of the FBG sensor was $\lambda_{B}=1523.67\;\mathrm{nm}$ at the initial state. A series of experimental tests were conducted to validate the proposed methodology. The measurement is relied on the detection of the Bragg wavelength shift. The measuring sensitivity highly depends on the resolution of optical spectrum analyzer (OSA). In this work, the resolution of the OSA (AQ 6331, Ando) is 0.02 nm. Thus, the strain and temperature sensitivities of this work are 20 με and 2 °C, respectively.

### 3.1. Temperature Measurement

**Figure 2:**
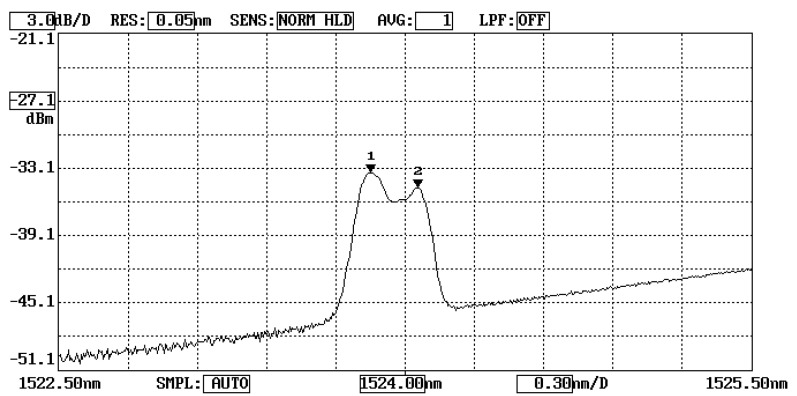
Optical spectrum reflected from the unbonded and bonded FBG due to a temperature of ΔT = 28.2 °C.

**Figure 3:**
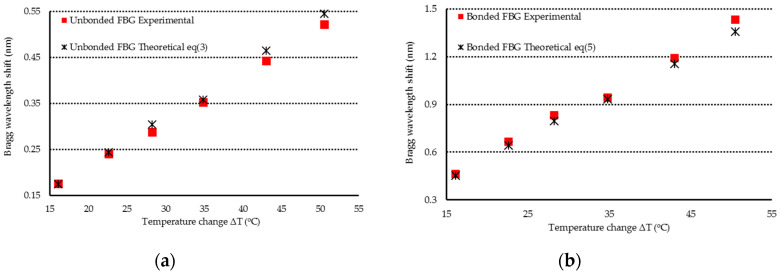
Bragg wavelength shift of unbonded and bonded FBG due to the temperature change. (**a**) Bragg wavelength shift of unbonded FBG (**b**) Bragg wavelength shift of bonded FBG.

The Al test specimen was placed in a thermal chamber. Temperature was varied from room temperature to 100 °C. A thermocouple near by the FBG sensor was used to record the temperature change. Figure 2 shows the optical spectrum reflected from the FBG sensor due to a temperature change of ΔT = 28.2 °C. The first and second peaks are associated with the Bragg wavelengths reflected from the unbonded and bonded parts of the FBG sensor, respectively. The Bragg wavelength shifts for the unbonded and bonded parts of FBG sensor can be evaluated using Equations (3) and (5), respectively. Experimental measurements of the Bragg wavelength shift were compared with the theoretical predictions as listed in Table 2. The comparisons between the theoretical prediction and experimental measurement of the Bragg wavelength shifts for unbonded FBG and bonded FBG are shown in Figure 3. It can be observed that the experimental measurements of the Bragg wavelength shifts for both unbonded and bonded FBG due to the temperature change are in close agreement with the theoretical predictions with difference less than 6%.

### 3.2. Strain Measurement

**Figure 4:**
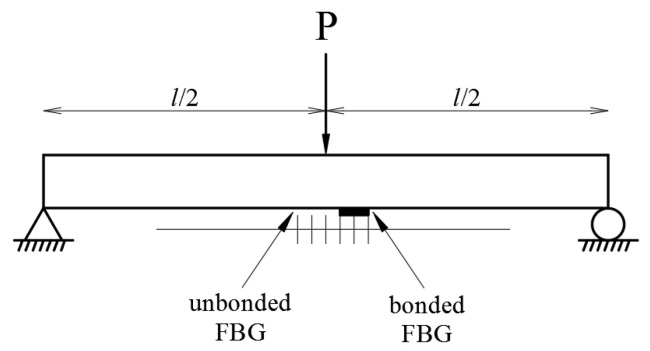
Three-point bending test on an Al specimen with bonded and unbonded FBG.

In the experimental tests, the Al specimen was subjected to a three-point bending test. The loading was applied along the y-direction, i.e., normal to the test specimen as shown in Figure 4. The bending strain of the Al specimen along the x-direction is given by:

(9) $$\varepsilon =\frac{Pxh}{4EI}\;\;\;\;\;\;\;\;\;\;\;\;\;\;\;\;\;0\leq x\leq l/2$$

$$\varepsilon =\frac{Ph}{4EI}\left(l-x\right)\;\;\;\;\;\;\;\;\;\;\;l/2\leq x\leq l$$

where *P* is the loading applied on the specimen and *E, h, l* and *I* are the Young’s modulus, thickness, length and moment of inertia of the Al specimen, respectively.

The theoretical prediction of the strain transferred from the specimen to the bonded FBG is calculated by substituting Equation (9) into Equation (6). The experimental measurement of the strain of the FBG bonded on the specimen is evaluated by substituting the measured Bragg wavelength shift $\Delta \lambda_{B}$ into Equation (2), which yields:

(10) $$\varepsilon_{M}=\frac{\Delta \lambda_{B}}{\lambda_{B}}{\left\{1-\frac{1}{2}n_{0}^{2}\left[\left(1-\upsilon_{f}\right)p_{12}-\upsilon_{f}p_{11}\right]\right\}}^{-1}$$

**Figure 5:**
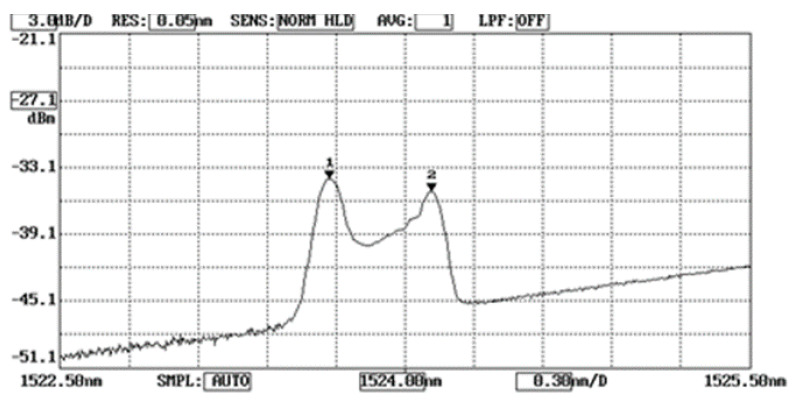
Optical spectrum reflected from the unbonded and bonded FBG due to a load of 102 N in the three-point bending test.

**Figure 6:**
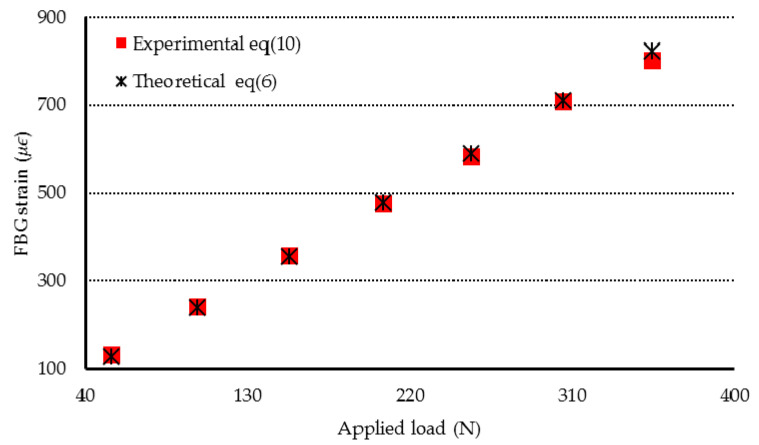
Strain transferred from the Al specimen to the bonded FBG in the three-point bending test with various loadings.

In the three-point bending tests, the loading was increasing from 0 to 350 N. As the test specimen subjected to mechanical loading, the mechanical strain was transferred from the test specimen to the bonded FBG. However, the unbonded FBG was not affected by the mechanical strain and it remained strain free. Therefore, the Bragg wavelength reflected from the FBG sensor was split into two peaks that correspond to the Bragg wavelengths reflected from the bonded and unbonded FBG, respectively. Figure 5 shows the optical spectrum reflected from the FBG sensor at the applied load of 102 N. The first and second peaks are associated with the Bragg wavelengths reflected from the unbonded and bonded parts of FBG sensor, respectively. The Bragg wavelength shift is equal to the difference of the Bragg wavelengths between the two peaks. Experimental measurements of the mechanical strain transferred from the specimen to the bonded FBG were compared with the theoretical predictions as listed in Table 3 and plotted in Figure 6. A good agreement is achieved between the experimental measurement and theoretical prediction of the strain of the bonded FBG sensor with difference less than 4%.

### 3.3. Simultaneous Measurement of Temperature and Strain

In this work, mechanical and thermal loadings were provided by a universal testing machine (H10KS, Hounsfield Test Equipment Ltd., Surrey, UK). The universal testing machine is equipped with a thermal chamber that is capable of heating a test specimen up to 270 °C. The Al specimen was placed in the thermal chamber and subjected to a three-point bending. In the experimental test, a specific temperature was set in the thermal chamber, followed by a mechanical loading on the test specimen through a three-point bending fixture. The Bragg wavelength reflected from the bonded FBG can be affected by both the temperature change and mechanical strain as shown in Equation (8). The effects of temperature and strain on the bonded FBG were taken into account for both theoretical prediction and experimental measurement. Thus, the bonded FBG cannot be used to measure the temperature. In the simultaneous measurement of temperature and strain, unbonded FBG was used to measure the temperature as shown in Equation (3), since it was not affected by the mechanical strain. Once the temperature was measured by the unbonded FBG. The mechanical strain can be determined from the bonded FBG by substituting the temperature change into Equation (8).

Utilizing the Bragg wavelength shift of the unbonded FBG as shown in Equation (3), the temperature change can be determined as follows:

(11) $$△T=\frac{\Delta \lambda_{B}}{K_{T}\lambda_{B}}$$

The Bragg wavelength shift of the bonded FBG can be used to evaluate the mechanical strain of the specimen from Equations (6) and (8) and written as follows:

(12) $$\varepsilon_{0}=\left\{\frac{\Delta \lambda_{B}}{\lambda_{B}}-\left[k_{\varepsilon }\varepsilon_{T}+k_{T}\Delta T\right]\right\}\frac{E_{f}\left(\frac{\pi r_{f}^{2}}{2hr_{p}E_{h}}+\frac{1}{E_{f}}\right)}{k_{\varepsilon }\left[1-\frac{cosh\left(\lambda_{1}x\right)}{cosh\left(\lambda_{1}L_{f}\right)}\right]}$$

**Figure 7:**
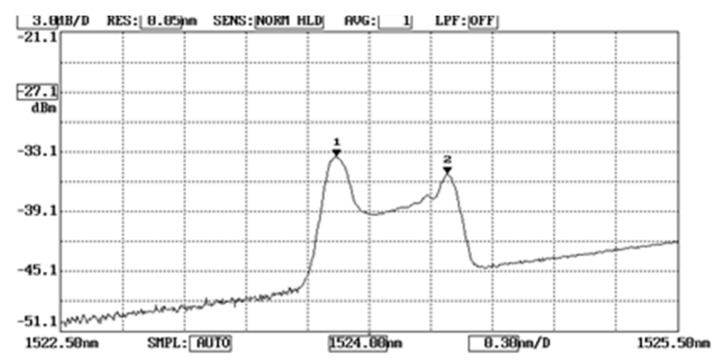
Optical spectrum reflected from the unbonded and bonded FBG subjected to a mechanical loading of 157 N at a temperature of 47 °C.

In the experimental tests, the temperature of the thermal chamber varied from room temperature to 100 °C while the mechanical loading was increased up to 350 N. Figure 7 shows the optical spectrum reflected from the FBG sensor as the Al specimen was subjected to a mechanical loading of 157 N at a temperature of 47 °C. The first and second peaks are associated with the Bragg wavelengths reflected from the unbonded and bonded parts of the FBG sensor, respectively. The temperature and mechanical strain of the Al specimen were determined by substituting the Bragg wavelength shifts of the unbonded FBG and bonded FBG into Equations (11) and (12), respectively. The temperature measured by FBG sensor was compared with the result from a thermocouple, while the mechanical strain of the Al specimen was validated with the theoretical prediction using Equation (9).

**Figure 8:**
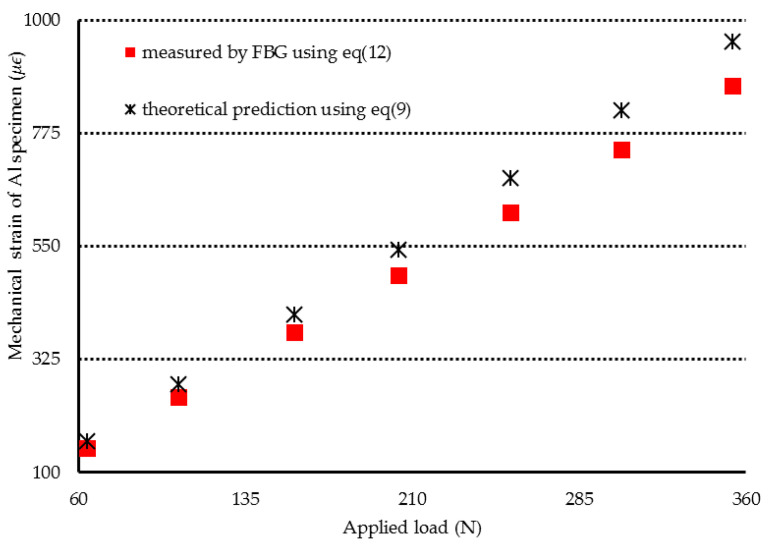
Mechanical strain of the Al specimen subjected to three-point bending with various loadings at a temperature of 47 °C.

**Figure 9:**
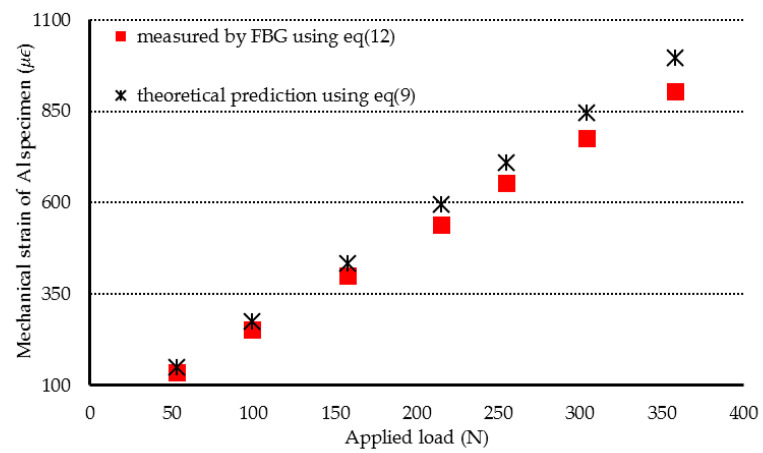
Mechanical strain of the Al specimen subjected to three-point bending with various loadings at the temperature of 78.1 °C.

**Figure 10:**
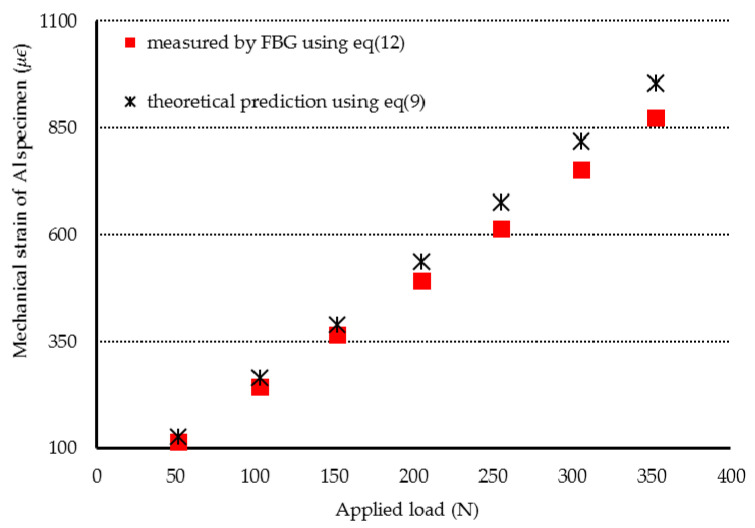
Mechanical strain of the Al specimen subjected to three-point bending with various loadings at a temperature of 96.5 °C.

In the first experimental test, the Al specimen was heated from 25.7 °C to 47 °C. The Bragg wavelength shift of $\Delta \lambda_{B}=0.232\;nm$ for the unbonded FBG was detected. Substituting $\Delta \lambda_{B}=0.232\;\mathrm{nm}$ into Equation (11) leads to the determination of a temperature of 45.2 °C for the Al specimen, which shows an error of 3.83% in comparison with the result measured by a thermocouple. Then, the Al specimen was subjected to a three-point bending under various loadings with the temperature kept at a constant of 47 °C. The mechanical strain of the Al specimen can be determined by substituting the Bragg wavelength shift of bonded FBG into Equation (12). The mechanical strains of the Al specimen measured by the FBG sensor were compared with the theoretical prediction Equation (9) as shown in Figure 8. Two more experimental tests were conducted on the Al specimen at temperatures of 75.8 °C and 96.5 °C, respectively. Figure 9 and Figure 10 show the FBG measurements and theoretical predictions Equation (9) of the mechanical strain for the Al specimen under various loadings with the temperature kept at a constant of 78.1 °C and 96.5 °C, respectively. It can be observed that simultaneous measurement of temperature and mechanical strain using an FBG sensor is in good agreement with the experimental results measured by thermocouple and theoretical prediction. This indicates that the discrimination between the temperature and strain can be realized using a single FBG sensor. The resolutions of temperature and strain for the present approach are 2 °C and 20 με, respectively. Alahbabi et al. [29] reported a temperature resolution of 6 °C and a strain resolution of 150 με using the anti-Stokes spontaneous Raman and Brillouin signals generated from a single light source. It demonstrates that the proposed technology using FBG sensor exhibited better resolutions of temperature and strain in comparison with the results reported by Alahbabi et al. [29] based on the Raman and Brillouin signals.

## 4. Conclusions

A simple and easy way to implement methodology based on the FBG sensing technology was proposed and experimentally demonstrated by the capability of measuring the temperature and mechanical strain simultaneously. It is well known that FBG sensor is sensitive to both the temperature and strain, resulting in an unwanted cross-sensitivity between these two parameters. In this work, half of the FBG sensor was bonded on the host structure, while the other half was left free. The host structure was an aluminum test specimen with dimensions of 20 × 3.8 × 0.5 ${\mathrm{cm}}^{3}$. Bragg wavelength reflected from the unbonded FBG can be utilized to measure the temperature and is independent of the strain, while the Bragg wavelength reflected from the bonded FBG allows the strain to be evaluated. Thus, the temperature and mechanical strain can be simultaneously measured using one single FBG sensor. Experimental results show that the errors of the measurements of temperature and mechanical strain using present approach are 6% and 4% in comparison with the thermocouple and theoretical prediction, respectively. Lv et al. [14] employed two FBG sensors to simultaneously measure the flow rate and temperature in a pipeline with temperature accuracy of ±1 °C in the range of 23–83 °C, which is closed to the present approach. A theoretical analysis verified by the experimental measurement illustrates that the proposed method with one single FBG sensor can be used to discriminate the temperature and strain.

## Figures and Tables

**Table 1:** Material properties of the optical fiber, protective coating, adhesive and host structure.

Young’s modulus of optical fiber *E_f_* = 72 GPa	radius of optical fiber $r_{f}=62.5$ μm
Young’s modulus of coating *E_p_* = 6.7 MPa	radius of coating $r_{p}=125$ μm
Young’s modulus of adhesive *E_a_* = 2 GPa	coefficient of thermal expansion of optical fiber $\alpha_{f}=0.5\;\mu \varepsilon /$°C
Young’s modulus of host structure *E_h_* = 72 GPa	coefficient of thermal expansion of coating $\alpha_{p}=76\;\mu \varepsilon /$°C
Poisson’s ratio of optical fiber $\nu_{f}$ = 0.17	coefficient of thermal expansion of adhesive $\alpha_{a}\;$= 20 με/°C
Poisson’s ratio of coating $\nu_{p}$ = 0.49	coefficient of thermal expansion of host structure $\alpha_{h}=23\;\mu \varepsilon /$°C
Poisson’s ratio of adhesive $\nu_{a}$ =0.4	gap $b/r_{p}$ = 0.2
Poisson’s ratio of host structure $\nu_{h}$ = 0.3	refractive index of optical fiber $n_{0}=1.456$
shear modulus of coating $G_{p}=2.2\;\mathrm{MPa}$	Pockel’s constant *P*_11_ = 0.121
shear modulus of adhesive *G_a_* = 0.714 MPa	Pockel’s constant *P*_12_ = 0.27
thermo-optic coefficient $\xi =1.4\times 10^{-5}/$°C	

**Table 2:** Bragg wavelength shifts for the unbonded and bonded parts of FBG sensor due to the temperature change.

Temperature Change ΔT (°C)	Bragg Wavelength Shift for the Unbonded FBG Δ$\lambda_{B}$ (nm)			Bragg Wavelength Shift for the Bonded FBG Δ$\lambda_{B}$ (nm)		
	Experimental Measurement	Theoretical Prediction Equation (3)	Error %	Experimental Measurement	Theoretical Prediction Equation (5)	Error %
16.1	0.176	0.174	1.15	0.464	0.452	2.65
22.6	0.240	0.244	1.64	0.664	0.637	3.59
28.2	0.288	0.304	5.26	0.832	0.797	4.39
34.8	0.352	0.358	1.68	0.944	0.934	1.07
43	0.442	0.464	4.74	1.192	1.155	3.20
50.5	0.522	0.545	4.22	1.432	1.356	5.61

**Table 3:** Strain transferred from the specimen to the bonded FBG sensor in the three-point bending tests.

Applied Load (N)	Specimen Strain $\varepsilon_{0}\;\left(\mu \epsilon \right)$ Equation (9)	Bragg Wavelength Shift Δ$\lambda_{B}$ (nm)	Experimental Measurement FBG Strain $\varepsilon_{M}\;\left(\mu \epsilon \right)$ Equation (10)	Theoretical Prediction FBG Strain $\varepsilon_{M}\;\left(\mu \epsilon \right)$Equation (6)	Error %
54.3	155	0.144	132	127	3.94
102	292	0.264	241	238	1.26
153	437	0.392	358	356	0.56
205	586	0.52	475	478	0.63
254	727	0.64	585	592	1.18
305	872	0.776	709	711	0.28
354	1013	0.88	804	825	2.55

## References

[1] Du C., Dutta S., Kurup P., Yu T., Wang X. (2020). A review of railway infrastructure monitoring using fiber optic sensors. Sens. Actuators A.

[2] Leung C.K.Y., Wan K.T., Inaudi D., Bao X., Habel W., Zhou Z., Ou J., Ghandehari M., Wu H.C., Imai M. (2015). Review: Optical fiber sensors for civil engineering applications. Mater. Struct..

[3] Zhang X., Lu Y., Wang F., Liang H., Zhang Y. (2011). Development of fully-distributed fiber sensors based on Brillouin scattering. Photonic Sens..

[4] Bao X., Chen L. (2012). Recent progress in distributed fiber optic sensors. Sensors.

[5] Albert J., Shao S.L.Y., Caucheteur C. (2013). Tilted fiber Bragg grating sensors. Laser Photonics Rev..

[6] Wu N., Zou X., Tian Y., Fitek J., Maffeo M., Niezrecki C., Chen J., Wang X. (2012). An ultra-fast fiber optic pressure sensor for blast event measurements. Meas. Sci. Technol..

[7] Li L., Xia L., Xie Z., Liu D. (2012). All-fiber Mach-Zehnder interferometers for sensing applications. Opt. Express.

[8] Grooteman F. (2020). Multiple load path damage detection with optical fiber Bragg grating sensors. Struct. Health Monit..

[9] Yu F., Okabe Y. (2020). Linear damage localization in CFRP laminates using one single fiber-optic Bragg grating acoustic emission sensor. Compos. Struct..

[10] Jacobsz S.W., Jahnke S.I. (2019). Leak detection on water pipelines in unsaturated ground by discrete fibre optic sensing. Struct. Health Monit..

[11] Tsukada T., Minakuchi S., Takeda N. (2019). Identification of process-induced residual stress/strain distribution in thick thermoplastic composites based on in situ strain monitoring using optical fiber sensors. J. Compos. Mater..

[12] Jang B.-W., Kim C.-G. (2019). Impact localization of composite stiffened panel with triangulation method using normalized magnitudes of fiber optic sensor signals. Compos. Struct..

[13] Anastasopoulos D., De Smedt M., Vandewalle L., De Roeck G., Reynders E.P.B. (2018). Damage identification using modal strains identified from operational fiber-optic Bragg grating data. Struct. Health Monit..

[14] Lv R.-Q., Zheng H.-K., Zhao Y., Gu Y.-F. (2018). An optical fiber sensor for simultaneous measurement of flow rate and temperature in the pipeline. Opt. Fiber Technol..

[15] Torres Górriz B., Payá-Zaforteza I., Calderón García P.A., Sales Maicas S. (2017). New fiber optic sensor for monitoring temperatures in concrete structures during fires. Sens. Actuators A.

[16] Weisz-Patrault D., Maurin L., Legrand N., Salem A.B., Bengrir A.A. (2015). Experimental evaluation of contact stress during cold rolling process with optical fiber Bragg gratings sensors measurements and fast inverse method. J. Mater. Process. Technol..

[17] Koyamada Y., Imahama M., Kubota K., Hogari K. (2009). Fiber-optic distributed strain and temperature sensing with very high measurand resolution over long range using coherent OTDR. J. Lightw. Technol..

[18] Madsen S.F., Carloni L. Lightning exposure of carbon fiber composites. Wind turbine blades. Proceedings of the 24th Nordic Insulation Symposium on Materials, Components and Diagnostics.

[19] Zaidi F., Nannipieri T., Soto M.A., Signorini A., Bolognini G., Pasquale F.D. (2012). Integrated hybrid Raman/fiber Bragg grating interrogation scheme for distributed temperature and point dynamic strain measurements. Appl. Opt..

[20] Horiguchi T., Shimizu K., Kurashima T., Tateda M., Koyamada Y. (1995). Development of a distributed sensing technique using Brillouin scattering. J. Lightw. Technol..

[21] Bolognini G., Soto M.A., Di Pasquale F. (2009). Fiber-optic distributed sensor based on hybrid Raman and Brillouin scattering employing multi-wavelength Fabry-Pérot lasers. IEEE Photon. Technol. Lett..

[22] Zou W., He Z., Hotate K. (2009). Complete discrimination of strain and temperature using Brillouin frequency shift and birefringence in a polarization-maintaining fiber. Opt. Express.

[23] Lu X., Soto M.A., Thévenaz L. (2017). Temperature-strain discrimination in distributed optical fiber sensing using phase-sensitive optical time-domain reflectometry. Opt. Express.

[24] Zhu P., Xie X., Sun X., Soto M.A. (2019). Distributed modular temperature-strain sensor based on optical fiber embedded in laminated composites. Compos. Part B.

[25] Kersey A.D., Davis M.A., Patrick H.J., LeBlanc M., Koo K.P., Askins C.G., Putnam M.A., Friebele E.J. (1997). Fiber grating sensors. J. Lightw. Technol..

[26] Tosi D. (2017). Review and Analysis of peak tracking techniques for fiber Bragg grating sensors. Sensors.

[27] Her S.-C., Huang S.-Y. (2016). Thermal Strain Measured by Fiber Bragg Grating Sensors. Sens. Mater..

[28] Her S.-C., Huang C.-Y. (2011). Effect of Coating on the Strain Transfer of Optical Fiber Sensors. Sensors.

[29] Alahbabi M.N., Cho Y.T., Newson T.P. (2005). Simultaneous temperature and strain measurement with combined spontaneous Raman and Brillouin scattering. Opt. Lett..

